# Exploring Thermally Conductive and Form-Stable Phase Change Composites: A Review of Recent Advances and Thermal Energy Applications

**DOI:** 10.3390/ma19061156

**Published:** 2026-03-16

**Authors:** Hong Guo, Boyang Hu, Huiting Shan, Xiao Yang

**Affiliations:** 1 Department of Energy and Chemical Engineering, Tianjin Ren’ai College, Tianjin 301636, China; 2 Carbon Neutrality Interdisciplinary Science Centre, College of Environmental Science and Engineering, Nankai University, Tianjin 300350, China; 3 Department of Chemical and Biomolecular Engineering, National University of Singapore, Singapore 117585, Singapore; 4 Joint School of National University of Singapore and Tianjin University, International Campus of Tianjin University, Binhai New City, Fuzhou 350207, China; 5 Hangzhou Huadian Huayuan Environment Engineering Co., Ltd., Hangzhou 310030, China

**Keywords:** phase change composites, thermal conductivity enhancement, thermal management, energy storage and conversion

## Abstract

**Highlights:**

- State-of-the-art advances of high-performance PCCs were reviewed.
- 3D aerogel-based skeletons are the most promising porous supporting materials.
- Multiple application prospects and future development directions were outlooked.

**Abstract:**

The global population explosion and accelerated industrialization have led to an increasing shortage of fossil fuels and environmental contamination, underscoring the urgent need to develop innovative energy storage technologies to improve energy utilization efficiency. As pivotal components in thermal energy storage (TES) systems, phase change materials (PCMs) enable spatiotemporal matching between thermal energy supply and demand through latent heat absorption and release during phase transitions. Organic PCMs are considered ideal candidates for thermal energy storage due to their high energy storage density, stable phase transition temperature, low supercooling, and negligible phase separation. However, inherent drawbacks such as low thermal conductivity, liquid leakage, limited light absorption, and lack of functionality have hindered their widespread application in advanced thermal management systems. Herein, we systematically summarize cutting-edge functionalization strategies for PCMs, progressing from conventional methods like thermal conductive particle blending and microencapsulation to the emerging design of 3D porous thermally conductive skeletons, including metal foams, boron nitride aerogels, carbon-based aerogels, and MXene aerogels. These frameworks not only enhance thermal transport via continuous conductive pathways and impart shape stability through capillary encapsulation but also, when integrated with photo-thermal, electro-thermal, and magneto-thermal conversion properties, enable broad applications in solar photo-thermal/photo-thermo-electric conversion, thermal management of electronics and batteries, building efficiency, and wearable thermal regulation. The review further addresses current challenges and future directions, highlighting scalable 3D framework fabrication, the shift to active thermal management, and innovative applications beyond conventional domains. By establishing a microstructure–property–application correlation, this work provides valuable insights for developing next-generation high-performance multifunctional phase change composites.

## 1. Introduction

Over the past several centuries, fossil fuels have served as the predominant energy source, underpinning the progress of human civilization. However, the growing depletion and non-renewable nature of fossil fuels have led to worldwide challenges such as resource scarcity, excessive energy waste, and a series of environmental pollution problems caused by energy consumption [1]. Renewable energy sources, including solar, geothermal, wind, biomass, and tidal energy, possess abundant reserves and sustainable power generation potential [2,3]. Nevertheless, their exploitation and utilization are generally constrained by weather conditions, time, and geographical limitations [4,5]. Emerging energy storage technologies provide effective solutions to the intermittency and volatility in energy transfer, thereby playing a crucial role in improving energy utilization efficiency.

Among the diverse landscape of energy storage solutions, thermal energy storage (TES) has emerged as a particularly attractive option. Thermal energy storage technology uses thermal storage materials as the medium to manipulate internal energy for heat redistribution, achieving a spatiotemporal match between thermal energy supply and demand [6,7]. This technology offers advantages such as high energy density, high storage efficiency, low cost, and potential for large-scale deployment. TES can be categorized into three main types: sensible heat storage, thermochemical storage, and latent heat storage [8,9]. Sensible heat storage achieves thermal energy storage by raising the temperature of a medium. This technology offers advantages such as simple operation, high safety, and excellent chemical and mechanical stability. However, it inherently causes the storage medium’s temperature to rise continuously during heat charging, making precise thermal regulation difficult. Moreover, its low volumetric energy storage density limits its applicability in many scenarios. Thermochemical heat storage, by contrast, relies on reversible chemical reactions to convert thermal energy into chemical energy and vice versa. Although it offers high energy storage density, its widespread adoption is hindered by challenges including stringent reaction conditions, complex operational processes, catalyst deactivation, high costs, and limited cycle life. In comparison, latent heat thermal energy storage (LHS), which utilizes the heat absorbed or released during phase transitions, presents a more advantageous alternative. It not only enables peak shaving and valley filling for thermal management but also features high energy storage density, stable operating temperatures, simple equipment, convenient operation, low cost for large-scale implementation, excellent chemical stability, and environmental friendliness [10,11]. Due to the near-isothermal behavior of phase change materials (PCMs) during energy storage and release, they effectively regulate the thermal balance between heat sources and the ambient environment, enabling accurate matching of thermal supply and demand in terms of timing, location, and intensity [12]. Currently, PCM-based latent heat storage technology has been widely applied in various fields, including energy conversion and storage systems [13,14], waste heat recovery [15], thermal management of electronics [16], battery thermal management [17], intelligent temperature control [18], and building energy conservation [19,20].

**Figure 1:**
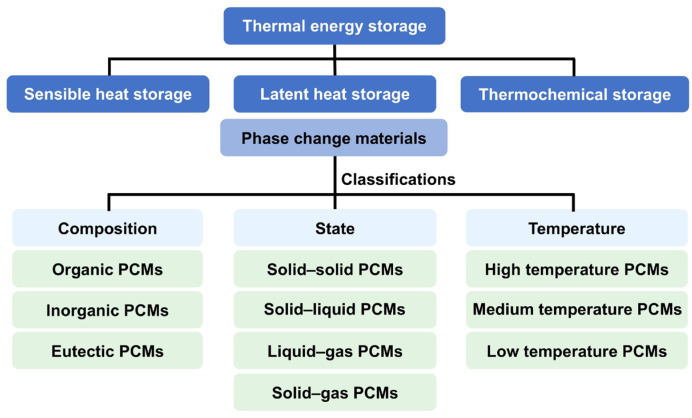
Different types of thermal storage technologies and classifications of PCMs.

PCMs exhibit considerable diversity in composition and properties [21], which can be systematically classified according to several criteria (Figure 1). (1) Based on the constituent elements, PCMs are categorized into organic, inorganic, and eutectic types. Inorganic PCMs encompass molten salts, hydrated salts, metals, alloys, and others; organic PCMs encompass fatty acids, aliphatic hydrocarbons, polyols, polyethers, and others; eutectic PCMs encompass inorganic–inorganic, organic–inorganic, and organic–organic eutectic PCMs. (2) According to the phase change temperature, PCMs are distinguished among high-temperature, medium-temperature, and low-temperature types. High-temperature PCMs include molten salts, metals, and alloys; medium-temperature PCMs include hydrated salts, aliphatic hydrocarbons, fatty acids, and polymers; low-temperature PCMs primarily consist of ice, hydrogels, water, and salts. (3) PCMs can also be categorized by their phase transition state including solid–solid, solid–liquid, liquid–gas, and solid–gas types. Solid–solid PCMs store and release thermal energy through crystalline phase transitions, offering advantages such as minimal volume change and the absence of liquid leakage. However, their thermal storage density is relatively low. Representative examples include polyols, cross-linked polymers, and inorganic salts. Solid–liquid PCMs, such as aliphatic hydrocarbons, fatty acids, hydrated salts, and molten salts, feature high thermal storage density and low cost. Their main limitation, however, is the risk of liquid leakage during operation. In contrast, liquid–gas and solid–gas PCMs suffer from encapsulation difficulties, demanding storage conditions, and leakage risks, which have so far restricted their practical application. When evaluated against key criteria such as energy storage density, operational cost, scalability, and safety, organic solid–liquid PCMs have garnered significant attention from both academic and industrial sectors [22,23] due to their high latent heat of fusion, stable phase transition temperature, minimal supercooling, and negligible phase separation. Despite these advantages, organic solid–liquid PCMs are inherently plagued by two major limitations: the low intrinsic thermal conductivity (<0.5 W·m^−1^·K^−1^) and liquid leakage risk [24,25]. These drawbacks substantially compromise the efficiency of heat transfer and storage, thereby restricting their practical deployment in advanced energy conversion/storage and thermal management systems.

Currently, intense research efforts have been focused on developing the strategies to enhance the thermal conductivity and form stability of PCMs [26,27,28]. On the one hand, PCMs are incorporated with high-thermal-conductivity fillers or encapsulated into shells to overcome the aforementioned defects. PCMs can be encapsulated in micron-sized spherical particles using organic, inorganic, or hybrid shells. This microencapsulation yields core–shell structures where the supporting shell prevents liquid leakage and maintains a solid macroscopic form during phase change. On the other hand, porous supporting substances are used to encapsulate PCMs, such as porous carbon, graphene foam, aerogel skeleton, etc. The porous adsorption strategy physically confines PCMs within a porous support through capillary action, yielding shape-stabilized composites that are resistant to liquid leakage. Porous materials are classified by pore diameter as microporous (<2 nm), mesoporous (2–50 nm), or macroporous (>50 nm). Pore size governs PCM encapsulation efficacy: excessively small pores hinder phase transitions, while overly large pores fail to contain the molten PCM. Therefore, mesopores and smaller macropores are preferred for preparing shape-stabilized composite PCMs. Furthermore, functional additives are tuned with conventional PCMs for expanding the application scenes of PCCs [29], including intelligent textile [30], infrared stealth [31], drug delivery [32], and solar/electric/magnetic-thermal energy conversion [33]. Although significant progress has been made in recent years, the advanced high-performance PCCs remains in infancy and require in-depth exploitation.

**Figure 2:**
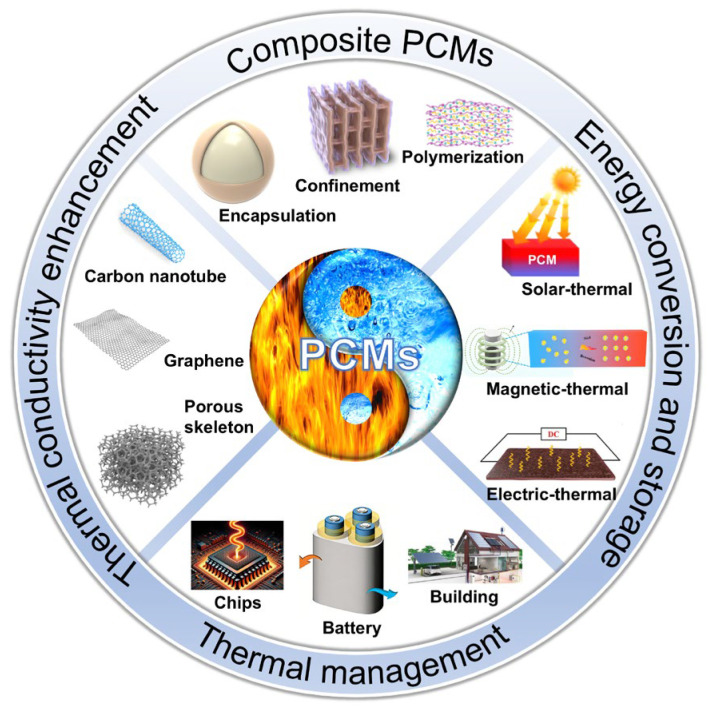
Integrated scheme of thermally conductive and form-stable PCCs, including fabrication strategies, thermal conductivity enhancement, and applications in thermal management and energy storage and conversion.

To date, existing reviews mainly focus on addressing either the morphological instability [34,35], low thermal conductivity of PCCs [34,36], or their applications in thermal management, energy storage, and energy conversion [37,38,39,40]. However, few reviews have comprehensively summarized the fabrication, thermal conductivity improvement, and advanced applications of PCCs in thermal management and energy harvesting. Different from Liu et al.’s [41] focus on functional fillers to endow PCMs with single/multiple energy conversion performance, this review focuses on the structural design of three-dimensional porous thermal conductive skeletons as the strategy to simultaneously solve the problems of low thermal conductivity and leakage of PCMs. The objective of this study is to offer a timely, thorough, and in-depth insight into the latest advances in the fabrication of thermally conductive and form-stable PCCs (Figure 2). Furthermore, the state-of-the-art applications of high-performance PCCs, including thermal management, energy storage, and energy conversion, are elaborated, while current challenges as well as future perspectives are highlighted. Therefore, we aim to furnish profound insights into the interrelationships among material preparation, thermal properties, and thermal management applications, thereby better guiding the design and utilization of advanced thermally conductive and form-stable PCCs.

## 2. Methodology

A systematic literature review was conducted following the Preferred Reporting Items for Systematic Reviews and Meta-Analyses (PRISMA) guidelines to evaluate the thermal conductivity enhancement and form stability of PCCs in thermal energy applications and identify the associated opportunities and challenges.

### 2.1. Data Sources and Search Strategy

The literature search was performed using the databases Scopus and Web of Science. These databases were selected due to their extensive coverage of peer-reviewed journals in materials science and thermal energy utilization, ensuring access to relevant studies. The search focused primarily on studies published between 2021 and 2026 to capture recent advancements in the PCC technology field, given the rapid development in this field over the past decade. Nonetheless, studies published prior to 2021 were also included if they provided valuable insights into PCC science and behavior.

The keywords were selected based on the research objectives that included understanding PCC properties, thermal conductivity enhancement, form stability and challenges in thermal energy applications. An iterative process was employed to refine the keywords, starting with broad terms like “phase change composites” and “thermal conductivity” and, after that, adding specific terms like “thermal energy storage”, and “form stability” based on preliminary search results. The Boolean search strings were built as follows:

- Scopus: (“phase change composite” OR “phase change material”) AND (“thermal conductivity” OR “thermal energy storage” OR “heat transfer” OR “latent heat” OR “thermal performance”) AND (“form stability” OR “shape stability” OR “encapsulation”).
- Web of Science: (“phase change composite” OR “phase change material”) AND (“thermal conductivity” OR “thermal energy storage” OR “heat transfer” OR “latent heat” OR “thermal performance”) AND (“form stability” OR “shape stability” OR “encapsulation”).

### 2.2. Inclusion/Exclusion Criteria

The studies were included in the review if they addressed the following:

- PCC integration into thermal management for electronic devices, battery thermal management, building energy conservation, wearable thermal management, solar-thermal energy conversion and storage, solar-thermo-electric energy conversion and storage, electro-thermal energy conversion and storage, and magneto-thermal energy conversion and storage.
- Thermal performance metrics exposing heat flux, thermal conductivity, latent heat, and thermal comfort.
- Quantitative/qualitative data on comparing PCC-integrated systems.
- Publication in peer-reviewed journals or conference proceedings.

The papers were excluded if they met the following criteria:

- They did not provide quantitative/qualitative data on PCC performance.
- They were not peer-reviewed, were not technical reports, or were non-academic publications.

## 3. Thermal Conduction Mechanism

Most commercially used PCMs for thermal energy storage, such as metals, inorganic salts, and paraffins, are solids prior to phase transition. These materials achieve reversible thermal energy storage and release primarily through transitions between ordered and disordered molecular structures. In solid materials, the thermal conduction mechanism generally falls into three categories: electronic heat conduction, phonon heat conduction, and photon heat conduction. These mechanisms are mediated by free electrons, lattice vibrations of phonons, and electromagnetic radiation, respectively. Among them, photon conduction plays a relatively minor role in most substances, as it relies on radiative transfer. Consequently, research has predominantly focused on the electronic and phonon thermal conduction mechanisms [42].

In metallic materials, heat conduction is governed primarily by the interactions and collisions among free electrons. These free electrons serve as both electrical and thermal charge carriers; therefore, the thermal conductivity is intrinsically linked to electrical conductivity. This relationship is quantitatively described by the Wiedemann–Franz law, which states that *k* = *LσT*, where *L* is the Lorentz number, *σ* is the electrical conductivity, and *T* is the absolute temperature [42]. Owing to this electronic heat conduction mechanism, metallic PCMs typically exhibit exceptionally high thermal conductivity, such as silver displaying a thermal conductivity of 429 W m^−1^ K^−1^.

In nonmetallic materials, which lack free electrons, heat conduction is governed primarily by lattice vibration waves. According to quantum theory, the energy of these lattice vibrations is quantized. Each quantum of vibrational energy is termed a phonon, which represents a collective excitation of atoms in a periodic lattice. The interaction between lattice vibration waves and the material can be understood as phonon scattering processes, through which thermal energy is transported. Specifically, phonons propagate by colliding with other phonons, grain boundaries, lattice defects, and other microstructural features [42]. From the perspective of kinetic theory, the thermal conductivity due to phonons can be expressed as: *k* = $\frac{1}{3}$*cvl*, where *c* is the heat capacity of the phonon per unit volume, *v* is the average velocity of phonon motion, and *l* is the mean free path.

It is important to note that the composition and morphology of PCMs and their composites are critical factors governing their thermal conduction mechanisms. As a result, no single theoretical model can adequately explain the thermal behavior of all PCM systems. In recent years, the diffusion-driven model has gained increasing recognition as a complementary mechanism to phonon conduction, particularly in highly defective crystals and amorphous materials. Thus, these developments highlight the need for continued research into the fundamental thermal transport mechanisms in PCMs.

## 4. Fabrication of Thermally Conductive and Form-Stable PCCs

### 4.1. Incorporation of Fillers

As previously noted, the inherently low thermal conductivity (<0.5 W·m^−1^·K^−1^) is a major obstacle hindering the practical applications of PCMs, limiting the thermal transport rate during thermal energy charging/discharging processes [21]. A primary solution to this fundamental constraint involves compounding high-thermal-conductivity fillers with the PCMs matrix. The fillers act as efficient phonon transmission pathways to improve the thermal conductivity of PCCs [43]. These high-thermal-conductivity fillers include metal, carbon-based materials, and ceramic fillers. For instance, Lin et al. [44] reported a 46.3% enhancement in the thermal conductivity of paraffin wax (PW) composite with the incorporation of merely 2.0 wt% copper (Cu) nanoparticles, which also served as nucleating agents to mitigate supercooling effects. Similarly, Wang et al. [45] achieved a 30.3% improvement in thermal conductivity by doping PW with 3.0 wt% carbon nanotubes (CNTs), albeit with an 8.9% reduction in melting enthalpy. In a comparative study, Sunden et al. [46] evaluated the efficacy of various nanoparticles (e.g., CuO, Al_2_O_3_, Fe_3_O_4_) in enhancing the thermal properties of PW, utilizing Span 80 as a dispersant to suppress agglomeration. Their results indicated that a 1.2 wt% loading of CuO increased thermal conductivity by 24.9% while only marginally reducing the phase change enthalpy by 1.5%. Further investigations by Yu et al. [47] on carbon-based nanofillers (CNTs, CNFs, GNPs) revealed a monotonic increase in composite thermal conductivity with filler content, accompanied by a gradual decline in energy storage density.

The direct blending method is favored for its procedural simplicity and cost-effectiveness. Nonetheless, the thermal enhancement is frequently constrained by pronounced interfacial thermal resistance at the filler-PCM boundaries, which impedes efficient phonon transport [48]. While employing high filler loading (exceeding 50 vol%) can substantially improve conductivity, it concurrently displaces a significant portion of PCM, resulting in a drastic deterioration of overall energy storage capacity of the composite [49]. Moreover, the tendency for particle agglomeration within the matrix further compromises heat-transfer efficiency and limits the attainable thermal conductivity of the PCCs [50]. Therefore, the pivotal challenge in fabricating high-performance, shape-stable PCCs is to engineer efficient thermal percolation networks at minimal filler concentrations, thereby maximizing thermal conductivity without excessively sacrificing the energy storage density of PCMs.

### 4.2. Micro/Nano Microencapsulation

Micro/nano microencapsulation has emerged as an effective strategy to address the leakage issue of solid–liquid PCMs [51,52]. This technique involves encapsulating PCM cores within a protective shell to form core–shell structured microcapsules, typically achieved through methods such as interfacial polymerization, in situ polymerization, emulsion polymerization, and sol–gel processes [53,54]. Based on shell composition, microcapsules are generally classified into three categories: organic shells (e.g., polyurethane, polyurea, poly(acrylic acid), melamine-formaldehyde resin), inorganic shells (e.g., SiO_2_, Al_2_O_3_, TiO_2_, ZnO), and carbon-based shells (e.g., GO, CNTs). Organic shells typically offer superior flexibility but suffer from relatively low thermal conductivity, whereas inorganic shells provide higher thermal conductivity, stronger rigidity, and greater mechanical strength [55].

Substantial research efforts have demonstrated the efficacy of this approach. For instance, Lai et al. [5] prepared a microencapsulated PCC containing a TiO_2_/Ti_2_O_3_ shell and n-Tetracosane core, exhibiting an excellent solar-thermal conversion efficiency of 93.7%, an improved thermal conductivity of 0.509 W·m^−1^·K^−1^, and a high latent heat of 144.5 J·g^−1^. When coupled with thermoelectric module, the multifunctional film achieved adaptive 24 h uninterrupted power generation, with 21.1 W m^−2^ output power density at 5000 W m^−2^ light intensity. Shekaari et al. [56] used an emulsion polymerization strategy to encapsulate ethanolamine-based ionic liquids within poly(acrylic acid) shells; the latent heat of microcapsules was as high as 369.755 J·g^−1^, offering efficient energy storage and showing strong potential for TES applications. Ran et al. [57] encapsulated tetradecane within a polyurethane acrylate shell via interfacial polymerization, obtaining microcapsules with a heat storage density of 112 J·g^−1^ and excellent cycling stability. Jiao et al. [58] reported PW microspheres with graphene oxide (GO) via interfacial self-assembly, followed by reduction to obtain reduced graphene oxide (rGO)/PW microcapsules. These composites exhibited a remarkable thermal conductivity enhancement, almost 58.5 times higher than pure PW, while maintaining high energy storage density and stability over 1500 thermal cycles.

A significant advantage of microencapsulation is its ability to impart additional functionalities to PCCs, such as thermochromic properties or mechanical resilience [59]. However, the technology faces inherent limitations: the shell material necessary for mechanical integrity inevitably reduces the overall heat storage density of the composite. Furthermore, the complex synthesis procedures and associated high manufacturing costs presently hinder its widespread large-scale application.

### 4.3. Porous Skeleton Encapsulation

The development of high-performance PCCs is significantly challenged by interfacial thermal resistances both at the junctions between adjacent conductive fillers and at the filler–PCM boundaries [60]. These resistances constitute major thermal barriers that limit the efficiency of heat-transfer pathways within the composite. To address this fundamental issue, the construction of porous skeletons has been proposed as an innovative strategy to achieve simultaneous enhancement in thermal conductivity and energy storage density [61]. The porous skeleton serves as an interconnected, continuous network that effectively bridges individual conductive fillers. This architecture significantly reduces the number of filler–filler junctions and decreases the total interfacial area between the fillers and the PCM matrix [28,62]. By minimizing these sources of thermal resistance, heat can be preferentially and efficiently transported along the high-conductivity pathways provided by the skeleton, enabling a synergistic improvement in thermal conductivity even at low filler loadings, thereby preserving a high overall energy storage capacity [63,64].

Furthermore, the intrinsic capillary forces and surface tension within the micro/nano-pores of the skeleton facilitate strong physical adsorption of the molten PCMs [65]. This mechanism effectively prevents liquid leakage and confers excellent shape stability of the PCCs. Owing to the dual benefits of superior thermal management and effective encapsulation, the fabrication of functional porous skeletons for PCM encapsulation has become a prominent research focus [66,67,68]. Representative 3D frameworks explored for this purpose include metal foams, boron nitride aerogels, carbon-based aerogels, and MXene aerogels.

#### 4.3.1. Metal Foams

Metal foams are a class of porous metallic materials characterized by a 3D interconnected network structure, which synergistically combines the intrinsic properties of metals (such as high thermal and electrical conductivity) with the lightweight and high specific surface area of porous materials [69,70,71,72]. Owing to these features, metal foams demonstrate high thermal conductivity, excellent electrical conductivity, high specific strength, and low density. Commonly metal-based 3D porous skeletons for PCM encapsulation include commercially available copper, nickel, and aluminum foams, as well as engineered nanostructures like silver and copper nanowire (CuNW) aerogels.

Research has consistently validated the effectiveness of these metallic skeletons. For instance, Wang et al. [73] encapsulated a synthesized ester-based cetyl palmitate (CP) within a nickel foam. The thermal conductivity of resulting Ni/CP composite was 4.86 times greater than that of pure CP while maintaining a high heat storage density of 180.9 J·g^−1^, alongside excellent thermal stability and reliability. Shang et al. [74] used nickel foam (NF) as substrate for impregnating PEG, and the NF/PEG realized an increased thermal conductivity of 1.58 W·m^−1^·K^−1^ and phase change enthalpy of 126.3 J·g^−1^, together with high thermal stability and superb photo-thermal conversion property. In a study focusing on nanoscale architectures, Feng et al. [75] prepared a self-supporting CuNW aerogel via a precursor route followed by supercritical drying and sintering. This aerogel effectively encapsulated PW, significantly enhancing both the thermal conductivity and shape stability of the composite. Remarkably, a minimal loading of 6.3 wt% CuNW aerogel resulted in a 130% increase in the thermal conductivity of PW. Huo et al. [76] prepared copper foam/palmitic acid composite by melting-vacuum impregnation, achieving a high thermal conductivity of 5.1 W·m^−1^·K^−1^ and a latent heat of 174.7 J·g^−1^, which were promising options of thermal energy storage mediums. Zhu et al. [77] utilized a sustainable pinewood template to create a biomorphic porous copper foam through a calcination and reduction process. The resulting copper foam/PW composite demonstrated outstanding leak-proof performance and achieved a vertical thermal conductivity of 6.7 W·m^−1^·K^−1^, which was 26.8 times that of pure PW, showcasing considerable potential for applications in thermal energy storage and management systems.

#### 4.3.2. Boron Nitride Aerogels

Boron nitride (BN), known as “white graphite”, exhibits a theoretical thermal conductivity of approximately 600 W·m^−1^·K^−1^, complemented by exceptional thermal stability and intrinsic electrical insulation properties [78]. The fabricated PCCs using BN-based aerogels thus uniquely combine high thermal conductivity with electrical insulation, rendering them particularly attractive for thermal management in scenarios requiring electrical safety, such as in modern electronics and electrical systems [79,80,81,82,83].

**Figure 3:**
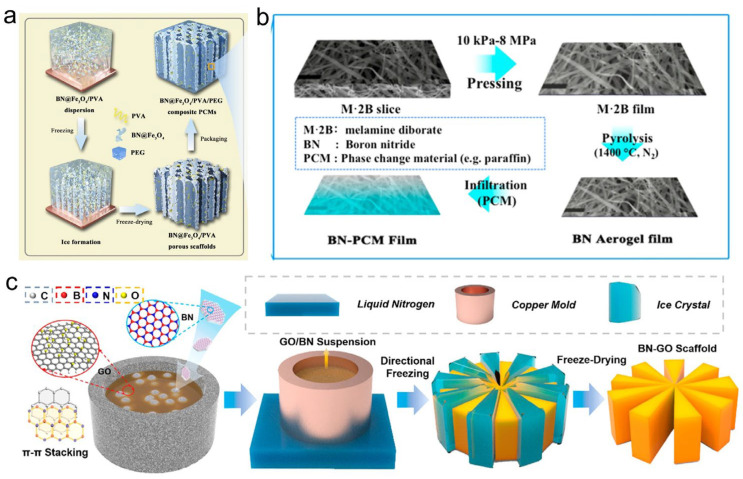
Schematic illustrations and performance characterization of PCCs based on functional BN aerogel skeletons. (**a**) PEG encapsulated with BN@Fe_3_O_4_/PVA aerogel [87]. (**b**) PW encapsulated with flexible BN aerogel film [88]. (**c**) PEG encapsulated within biaxially oriented BN/GO aerogel [89].

Significant progress has been made in the design of BN aerogel skeletons [84,85,86]. Yang et al. [87] engineered a hierarchical BN@Fe_3_O_4_/PVA skeleton by modifying BN nanosheets with Fe_3_O_4_ nanoparticles and assembling them via unidirectional freezing. After encapsulating polyethylene glycol (PEG), the composite achieved a thermal conductivity of 1.84 W·m^−1^·K^−1^ at a 25.4 wt% filler loading, alongside leak-proof performance and efficient photo/magnetic-thermal conversion capabilities (Figure 3a). Pursuing flexibility, Zhang et al. [88] developed a flexible BN aerogel through freeze-drying and thermal treatment. The resulting composite film after PW impregnation maintained excellent flexibility and electrical insulation, showing great potential for thermal management in flexible and portable electronics (Figure 3b). To optimize thermal pathways, Fu et al. [89] constructed a dually oriented BN/GO aerogel using BN as the primary building block and GO as a binder via radial freezing. The final BN/GO/PEG composite, with only 11.65 vol% BN, exhibited a high thermal conductivity of 2.94 W·m^−1^·K^−1^ and a heat storage density of 147.5 J·g^−1^ and achieved a power output of 40.28 W·m^−2^ when applied in a solar photo-thermal-electric conversion device (Figure 3c). For multifunctional systems, Yang et al. [90] designed a innovative bilayer porous skeleton using a two-step ice-templating method, featuring a top layer of black bacterial cellulose (BC) with thermochromic particles for light absorption and a bottom layer of white BC/BN for thermal conduction. After PEG encapsulation, this structure demonstrated excellent leak-proof performance, a high thermal conductivity of 3.26 W·m^−1^·K^−1^, and superior light absorption, indicating promising application in advanced solar energy conversion systems.

#### 4.3.3. Carbon-Based Aerogels

The preconstruction of 3D porous skeletons from carbon-based aerogels such as graphene [91,92], carbon nanotubes [93,94,95], carbon fibers [96], expanded graphite [97], and carbonized polymers [98,99], represents a highly prominent research direction for developing advanced PCCs. These carbon architectures provide excellent thermal conductivity, structural stability, and multi-functionality.

**Figure 4:**
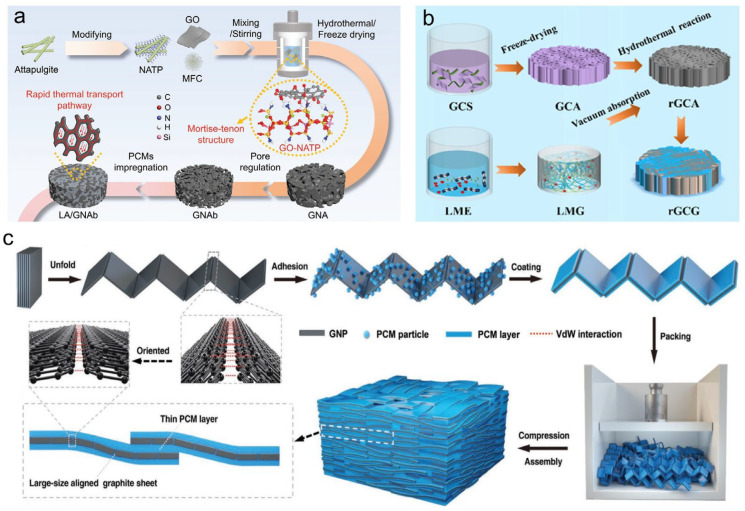
Schematic diagrams and properties of PCCs based on carbon aerogel skeletons. (**a**) Lauric acid encapsulated with amino-attapulgite/graphene hybrid aerogel [102]. (**b**) A rGO aerogel for encapsulating lauric acid/myristic acid eutectic mixture [104]. (**c**) PW confined within network of expanded graphite nanosheets [106].

Min et al. [100] constructed a honeycomb-structured graphite nanosheet/gelatin aerogel via directional freezing. After encapsulating PW, the composite achieved a high encapsulation ratio of 93.2 wt%, a thermal conductivity of 3.75 W·m^−1^·K^−1^, a heat storage density of 146.4 J·g^−1^, and desirable photo-responsive behavior, making it suitable for solar photo-thermal conversion. Liao et al. [101] developed a lightweight conductive skeleton by impregnating melamine foam (MF) with GO, followed by thermal reduction and carbonization, to obtain a graphene aerogel/MF (c-GA/MF) network for PEG encapsulation. The composite displayed a thermal conductivity of 1.32 W·m^−1^·K^−1^ and good shape stability. Tang et al. [102] prepared amino-attapulgite/graphene hybrid aerogels (GNA) for encapsulating lauric acid (LA), and the impregnated composite PCMs achieved a thermal conductivity of 1.164 W m^−1^ K^−1^ and effective encapsulation rate of 93.1% (Figure 4a). In efforts to further enhance thermal performance, Liu et al. [103] prepared a robust graphene/carbide aerogel using graphene nanoplatelets and aramid nanofibers as binders through unidirectional freeze-casting and carbonization. The resulting PEG-based composite reached a thermal conductivity of 4.85 W·m^−1^·K^−1^ and a latent heat of 149.7 J·g^−1^, showing promise for thermal energy storage and conversion systems. Chen et al. [104] fabricated a rGO aerogel through freeze-drying and hydrothermal methods, which was then vacuum-impregnated with a lauric acid/myristic acid eutectic mixture. The resulting composite demonstrated an exceptional PCM adsorption efficiency of 99.7%, a heat storage density of 124.6 J·g^−1^, and remarkable photo-thermal and electro-thermal conversion efficiencies of 96.5% and 82.3%, respectively, indicating strong potential for wearable thermal management (Figure 4b). Li et al. [105] fabricated carbon fiber (CF)-reinforced PEG composites via freeze-casting, achieving an ultrahigh thermal conductivity of 23.1 W·m^−1^·K^−1^ with 45 wt% CF loading. However, this came at the cost of a significant reduction in latent heat from 133.5 J·g^−1^ to 62.0 J·g^−1^, highlighting the typical trade-off between thermal conductivity and energy storage capacity. Wang et al. [106] employed a pressure-induced method to construct a large-scale, aligned graphite nanoplatelet network within stearic acid and PW matrices. With filler contents below 40 wt%, the PCCs exhibited widely tunable thermal conductivity (4.4–35.0 W·m^−1^·K^−1^) while maintaining excellent phase change behavior and leak-proof performance (Figure 4c).

**Figure 5:**
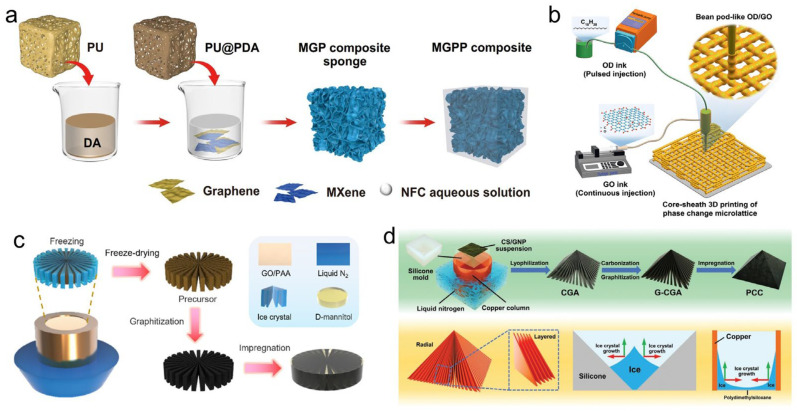
Graphene aerogels prepared by (**a**) soft template method [109], (**b**) 3D printing method [111], (**c**) radial freezing method [113], and (**d**) bidirectional freezing method [114].

Owing to exceptional intrinsic thermal conductivity of the graphene (theoretically up to ~5000 W·m^−1^·K^−1^), the use of graphene aerogels for encapsulating and thermally enhancing PCMs constitutes a major research thrust [107]. Fabrication strategies for these aerogels are diverse, encompassing chemical vapor deposition (CVD), hydrothermal assembly, soft templating, 3D printing, and ice-templating (freeze-casting) techniques [108]. Significant advances have been demonstrated across these methods. Jin et al. [109] constructed a 3D MXene/graphene hybrid aerogel using a soft-template and cyclic dip-coating approach. After PEG encapsulation, the composite delivered a thermal conductivity of 2.44 W·m^−1^·K^−1^, a heat storage density of 129.3 J·g^−1^, an electromagnetic interference shielding effectiveness of 43.3 dB, and mechanical strength four times greater than pure PEG (Figure 5a). Qi et al. [110] employed CVD to grow high-quality graphene foam (HGF) on a nickel template, subsequently impregnating it with PW. The thermal conductivity of resulting HGF/PW composite was 744% higher than pure PW while maintaining good shape stability, showing promise for solar photo-thermal conversion and storage. Drawing inspiration from nature, Yang et al. [111] fabricated pod-structured graphene/octadecane microlattices via 3D printing. The interconnected graphene network provided effective encapsulation, yielding a composite with a thermal conductivity of 1.67 W·m^−1^·K^−1^, an energy density of 190 J·g^−1^, and exceptional cycling stability (Figure 5b). Zhao et al. [112] utilized a hydrothermal method to regulate graphene oxide (GO) orientation, yielding a conical graphene aerogel (CGA) after graphitization. Upon encapsulating tetradecanol, the composite achieved a thermal conductivity of 4.54 W·m^−1^·K^−1^ and a high heat storage density of 206.1 J·g^−1^ with only 7.05 wt% filler, alongside excellent leak-proof performance and 84.0% solar photo-thermal efficiency. Focusing on structural control, Fu et al. [113] prepared biaxially oriented graphene aerogels via radial freezing of GO and polyacrylic acid (PAA), followed by graphitization. After impregnation with D-mannitol, the composite achieved a high thermal conductivity of 8.80 W·m^−1^·K^−1^, an energy storage density exceeding 190 J·g^−1^, and a solar photo-thermo-electric conversion efficiency of ~2.40% (Figure 5c). Zhao et al. [114] employed bidirectional freezing to fabricate pyramidal graphitized chitosan/graphene aerogels for PEG encapsulation. The composite exhibited a thermal conductivity of 2.90 W·m^−1^·K^−1^, an energy density of 178.8 J·g^−1^, leak-proof performance, and 90.4% solar thermal efficiency, reaching 99.7 °C under 200 mW·cm^−2^ irradiation (Figure 5d).

#### 4.3.4. MXene Aerogels

MXene, an emerging class of graphene-like two-dimensional materials composed of transition metal carbides/nitrides, has gained prominence as a functional filler for PCCs [115], owing to its near-theoretical photo-thermal conversion efficiency, exceptional electrical conductivity, and high intrinsic thermal conductivity [116]. The assembly of MXene into three-dimensional aerogel skeletons further extends these properties, enabling the development of PCCs with integrated functions.

**Figure 6:**
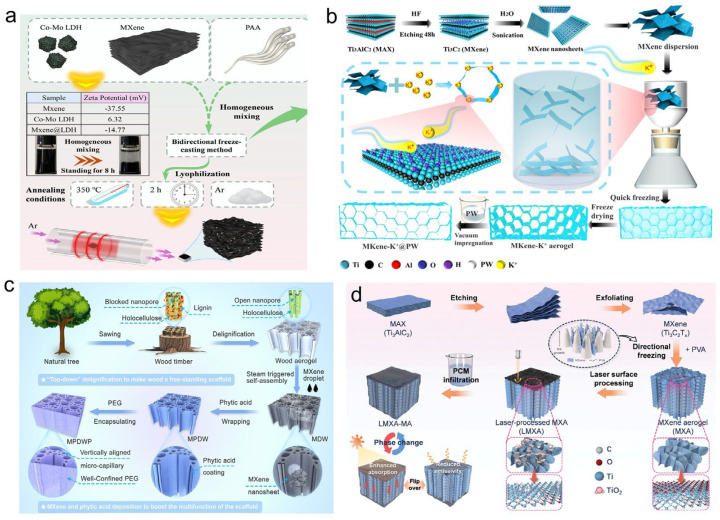
Schematic illustrations of multifunctional phase change composites based on MXene aerogel skeletons. (**a**) PW encapsulated with MXene/PI aerogel [117]. (**b**) PW encapsulated in a MXene/K^+^ aerogel [120]. (**c**) A biomimetic MXene/phytic acid-coated wood aerogel for PEG encapsulation [121]. (**d**) Myristic acid encapsulated within an anisotropic MXene-based aerogel [123].

Feng et al. [117] percolated MXene networks and LDH thermal bridges in 3D hierarchical polyimide aerogels. After encapsulating paraffin, the composite exhibited a melting enthalpy of 131.6 J·g^−1^, with a loading rate of 91.5% (Figure 6a). Sheng et al. [118] fabricated MXene/polyimide (PI) aerogels through freeze-drying and thermal imidization, using MXene as the building block and polyamic acid as a binder. After vacuum impregnation with PEG, the composite demonstrated a high PCM loading capacity of 98.1%, an energy storage density of 167.9 J·g^−1^, and notable flame-retardant properties. Cao et al. [119] employed konjac glucomannan and cellulose nanocrystals as dispersants and structural aids to form MXene-based aerogels via freeze-drying, which were subsequently carbonized. The resulting MXene/biocarbon skeleton, after encapsulating PW, exhibited an energy storage density of 215.7 J·g^−1^ and an EMI shielding effectiveness of 45.0 dB, alongside robust shape stability and cycling durability.

To enhance structural control, Qu et al. [120] introduced potassium ions (K^+^) to induce MXene self-assembly, creating MXene/K^+^ aerogels through vacuum filtration and freeze-drying. The composite with PW achieved an outstanding energy storage density of 261.7 J·g^−1^, a high EMI shielding effectiveness of 57.7 dB, and a photo-thermal conversion efficiency of 98.4% (Figure 6b). Chen et al. [121] developed a biomimetic approach by coating a wood-derived aerogel with phytic acid (PA) and MXene via evaporation-induced self-assembly prior to PEG encapsulation. The final composite offered a balanced performance with a thermal conductivity of 0.82 W·m^−1^·K^−1^, an energy storage density of 135.5 J·g^−1^, an EMI shielding effectiveness of 44.5 dB, and a photo-thermal efficiency of 98.6%, complemented by flame-retardant and leak-proof capabilities (Figure 6c). Ye et al. [122] constructed a hybrid MXene/sodium alginate/carbon nanotube aerogel via freeze-drying. After impregnation with tetradecylamine, the PCC demonstrated a high energy storage density of 217.8 J·g^−1^, an encapsulation efficiency of 91.0%, and excellent mechanical properties coupled with cyclic thermal stability. Zhu et al. [123] prepared a surface-engineered anisotropic MXene-based aerogel (LMXA), and the composite PCMs were produced after integrating with myristic acid (MA), which exhibited a thermal energy storage of 192.4 J·g^−1^ and encapsulation rate of 94.4% (Figure 6d).

### 4.4. Alternative Strategies

Beyond the mainstream strategies of thermal conductive filler blending, microencapsulation, and porous skeleton encapsulation, innovative functionalization approaches for PCMs continue to emerge. These include advanced techniques such as graft copolymerization [124], hybrid sintering, electrospinning [125], fiber coating, and 3D printing [126], which offer unique pathways to tailor the properties and forms of PCCs for specialized applications. For instance, beyond physical blending, Liu et al. [127] developed novel solid–solid PCCs by covalently grafting PEG onto a thermosetting phenolic resin backbone. The work demonstrated that the phase transition temperature of the resulting PCCs could be precisely tuned by varying the molecular weight of the grafted PEG chains, providing a molecular-level strategy for customizing thermal properties. Li et al. [128] employed a hybrid sintering method, where diatomite was uniformly blended with PEG, LiNO_3_, and Na_2_SO_4_ in varying ratios. The subsequent sintering treatment yielded shape-stabilized PCCs, leveraging the synergistic effects of the composite constituents. Shifting towards application-oriented forms, Xiang et al. [129] fabricated core–sheath-structured phase change fibers via melt-spinning of modified PEG and polycaprolactamide mixtures. This approach demonstrates significant potential for integrating active thermal management functionality directly into wearable smart textiles. Further advancing fiber-based systems, Huang et al. [130] engineered composite phase change fibers through coaxial electrospinning, using thermoplastic polyurethane as the sheath to encapsulate a PEG core. A subsequent electrostatic spraying of BN particles followed by hot-pressing enabled the fabrication of flexible phase change membranes that successfully integrated flame retardancy, excellent ductility, and high thermal conductivity.

The Table 1 summarizes the fabrication methods and key performance metrics across the above representative PCCs, specifically the thermal conductivity and latent heat.

## 5. Applications of Thermally Conductive and Form-Stable PCCs

### 5.1. Thermal Management

The distinctive characteristics of advanced PCMs, including the temperature-regulating capability, rapid thermal response, and high energy storage density, render them highly effective for thermal management applications [131,132]. By storing and releasing latent heat, PCMs contribute significantly to maintaining the operational stability of equipment, improving energy efficiency, and enhancing human comfort. Consequently, PCM-based thermal management technologies have been extensively explored and applied in many fields, such as thermal management of electronic devices, temperature control for power batteries, building energy conservation, and personal wearable thermal regulation.

#### 5.1.1. Thermal Management for Electronic Devices

The ongoing trend towards higher integration, superior performance, and progressive miniaturization of chips and electronic components has resulted in substantially increased power densities and significant heat generation. The associated thermal management challenges directly threaten the operational stability and service life of electronic devices [133]. Thus, it creates an urgent demand for advanced thermal management materials. Organic PCMs are considered prime candidates for this role due to their high latent heat storage capacity and near-isothermal behavior during solid–liquid phase transitions, which enable efficient heat absorption and temperature stabilization [134].

**Figure 7:**
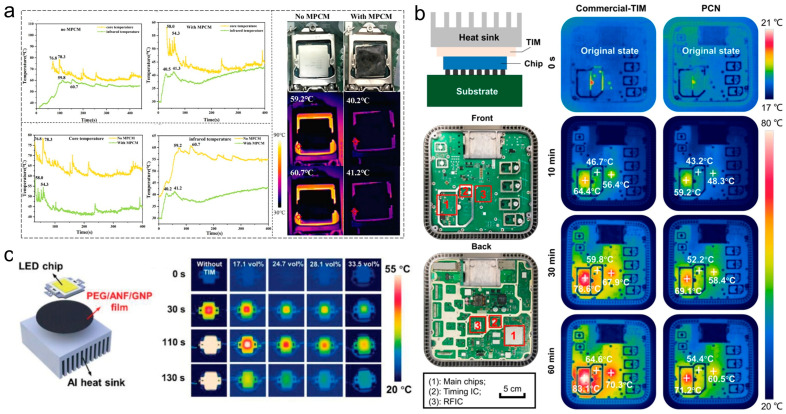
Application of high-performance PCCs in thermal management of electronic devices. (**a**) 3D MWCNT/CaCl_2_ 6H_2_O [135], (**b**) PEG@TPU/BNNS [130], (**c**) PEG/ANF/GNP [137].

Substantial research demonstrates their effectiveness in practical scenarios. Ma et al. [135] prepared align CNT/CaCl_2_·6H_2_O PCC by directional freezing, yielding an excellent thermal conductivity of 6.86 W·m^−1^·K^−1^. A 24 °C reduction in peak core temperature can be resulted when employing for the transient thermal management of CPUs (Figure 7a). Li et al. [105] fabricated carbon fiber (CF)/PEG composites via freeze-casting. With a 45.0 wt% CF loading, the composite achieved a high thermal conductivity of 23.1 W·m^−1^·K^−1^ and a latent heat of 62.0 J·g^−1^. When applied to dual-core CPUs, it effectively maintained operating temperatures within safe limits. Huang et al. [130] engineered flexible PEG@TPU/BN membranes via coaxial electrospinning and hot-pressing, achieving a thermal conductivity of 28.3 W·m^−1^·K^−1^ and a latent heat of 101 J·g^−1^. Deployed as a TIM between chips and heat sinks in 5G base stations, it eliminated “hot spots” and reduced chip temperatures by 18 °C compared to commercial TIMs (Figure 7b). Jiang et al. [136] fabricated polydimethylsiloxane (PDMS)/PW/BN composites using scraping, hot-pressing, and roll-cutting. With a 13.0 wt% BN loading, the composite reached a thermal conductivity of 2.16 W·m^−1^·K^−1^ and a latent heat of 81 J·g^−1^. Applied to 10 W LED chips, it lowered the saturation temperature by 10 °C relative to a commercial M100B15 material. Han et al. [137] developed laminated graphene nanoplatelet/aramid nanofiber/PEG phase change membranes through a sol–gel process. At 28.1 vol% graphene nanoplatelet loading, the membrane exhibited a thermal conductivity of 23.7 W·m^−1^·K^−1^ and a latent heat of 76.3 J·g^−1^. Used as a thermal interface material (TIM) on LED chips, it reduced the saturation temperature by 42.8 °C compared to operation without a TIM (Figure 7c).

#### 5.1.2. Battery Thermal Management

The operating temperature is a critical factor governing the performance of lithium-ion batteries, significantly influencing their capacity, charge/discharge efficiency, cyclic stability, service life, and safety [138,139]. Consequently, developing efficient thermal management systems is paramount for ensuring battery reliability and safety. High-thermal-conductivity PCCs are particularly promising for this application, as they integrate efficient heat storage and dissipation capabilities [140,141], enabling passive thermal regulation without external energy input.

**Figure 8:**
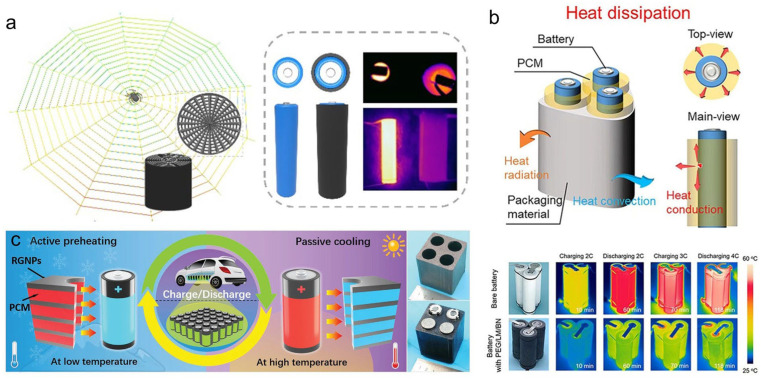
Application of PCCs in battery thermal management. (**a**) Graphene aerogel/paraffin wax (GS/PW) composite for battery encapsulation and thermal runaway mitigation [142]. (**b**) BN/liquid metal/polyethylene glycol (BN/LM/PEG) composite demonstrating anisotropic thermal conduction for reducing battery temperature rise [143]. (**c**) Expanded graphite/PEG@polyurethane (EG/PEG@PU) dual-encapsulated composite enabling both passive cooling and active heating functions [144].

Substantial research efforts have demonstrated the efficacy of various PCC designs. Huang et al. [142] fabricated a graphene aerogel (GS) via hydrothermal synthesis and radial freeze-casting, followed by impregnation with PW. The resulting GS/PW composite, with only 2.25 vol% GS loading, exhibited anisotropic thermal conductivities of 1.78 W·m^−1^·K^−1^ (in-plane) and 2.58 W·m^−1^·K^−1^ (through-plane), coupled with an energy storage density of 158.9 J·g^−1^. When used to encapsulate batteries, the composite effectively regulated temperature and mitigated thermal runaway risks (Figure 8a). Fu et al. [143] constructed a boron nitride/liquid metal (BN/LM) aerogel using radial freeze-casting and subsequently impregnated it with polyethylene glycol (PEG). The BN/LM/PEG composite achieved high thermal conductivities of 7.6 W·m^−1^·K^−1^ (in-plane) and 8.8 W·m^−1^·K^−1^ (through-plane), along with an energy density of 80 J·g^−1^. In thermal management tests for 18650 lithium-ion batteries, it reduced the maximum temperature rise by 10 °C (Figure 8b). Li et al. [144] developed dual-encapsulated EG/PEG@PU composites by infiltrating PEG-loaded PU into expanded graphite (EG). The material demonstrated exceptional thermal conductivity (27.0 W·m^−1^·K^−1^), electrical conductivity (51.0 S·cm^−1^), and energy density (150.4 J·g^−1^), satisfying both passive cooling and active heating requirements for battery thermal management (Figure 8c). Jia et al. [145] engineered a sulfur-bonded, crosslinked interpenetrating network of CNT and styrene–butadiene–styrene (SBS) for PW encapsulation. The composite delivered an energy density of 147.6 J·g^−1^, a tensile strength of 1.47 MPa, and excellent mechanical flexibility. Applied as a coating on battery surfaces, it effectively minimized capacity fade and enhanced safety by preventing thermal runaway.

#### 5.1.3. Building Energy Conservation

PCMs demonstrate significant potential for enhancing building energy efficiency through their integration into construction elements such as concrete, mortar, bricks, and gypsum boards [146]. By incorporating PCMs into wall linings, ceiling interlayers, and building envelopes, the latent heat storage and release characteristics can be leveraged to improve dynamic thermal regulation, smooth out indoor temperature fluctuations, and substantially reduce heating and cooling energy consumption in buildings [147,148].

Research has explored various integration strategies and material designs. Wang et al. [149] developed a zero-energy passive heating system based on solar thermal storage PCMs, which significantly reduced the building’s reliance on auxiliary energy for space heating. Feng et al. [150] prepared microencapsulated PCCs using a thermally expanded microsphere foaming technique with PW as the core material for filling wall cavities, which improved overall thermal insulation and enhanced occupant comfort. Sui et al. [151] engineered binary eutectic PCM gels with a high latent heat of 213.2 J·g^−1^ via an emulsion templating method. When integrated into building roofs, these gels effectively moderated indoor temperatures by absorbing solar heat during the day and releasing it during cooler night hours. Cao et al. [152] synthesized shape-stabilized organic/inorganic PCCs with a latent heat of 174.1 J·g^−1^, which offered the added advantages of flame retardancy and avoided issues of supercooling or phase separation, contributing to improved thermal comfort. Wang et al. [153] designed a biomimetic hierarchical thermal management device that integrated a PCM layer with a photo-thermal absorption layer and a silica aerogel insulation layer. This multi-functional system was able to raise indoor temperatures by up to 12.1 °C through efficient solar energy harvesting and storage, demonstrating particular promise for building heating in cold environments.

#### 5.1.4. Wearable Thermal Management

Maintaining the human core temperature within a narrow physiological range is crucial for ensuring metabolic stability and personal comfort. Organic PCMs present a promising avenue for developing zero-energy and passive thermal management solutions in wearable applications, owing to their high latent heat, tunable phase transition temperatures, biocompatibility, and excellent cycling stability [154,155]. However, inherent drawbacks such as leakage, rigidity, and low thermal conductivity pose significant challenges for their direct integration into textiles or flexible devices.

**Figure 9:**
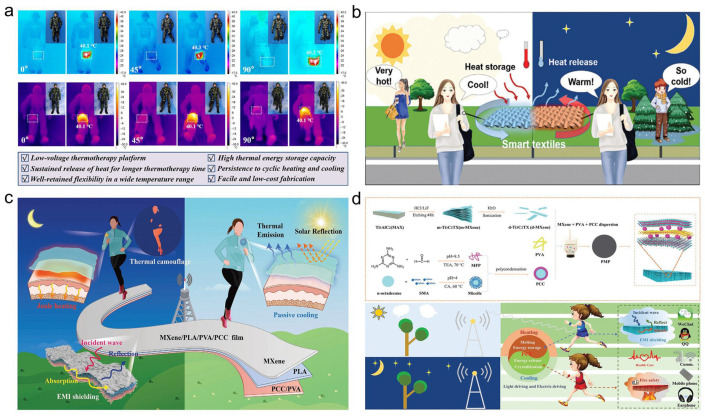
Application of advanced PCCs in wearable thermal management. (**a**) Flexible polypyrrole-modified melamine foam/PEG (PPy@MF-PEG) composite [156]. (**b**) Smart textile integrating silver nanowire cores with MXene/graphene sheaths (AgNWs/MXene/PEG) [158]. (**c**) Janus-type multifunctional PCC film enabling active/passive thermal regulation and EMI shielding [160]. (**d**) Biomimetic “brick-and-mortar” structured flexible PCC film for multi-source thermal management [161].

To overcome these limitations, advanced composite strategies have been employed. Chen et al. [156] developed flexible PCCs by encapsulating PEG within a polypyrrole-modified melamine foam skeleton. The resulting composite, with a latent heat of 150.1 J·g^−1^, not only provided passive thermal buffering but also demonstrated effective Joule heating capabilities for personal warming in cold environments (Figure 9a). Liu et al. [157] fabricated graphene–boron nitride (G-BN) hybrid fibers via wet-spinning, which were subsequently assembled into breathable, leakage-resistant nonwoven fabrics with a high latent heat of 206.0 J·g^−1^, suitable for use in smart masks and garments. Miao et al. [158] engineered smart textiles by constructing a core–sheath structure with silver nanowire (AgNW) cores and MXene-induced interlocked graphene sheaths, based on a similar compatibility principle. The textile exhibited exceptional dual-mode Joule heating and photo-thermal conversion effects, making it suitable for wearables under extreme conditions (Figure 9b). Shi et al. [159] developed flexible porous membranes composed of polyvinylidene fluoride (PVDF)/BN via a solvent exchange method. The membrane achieved a 200% enhancement in thermal conductivity compared to pure PW while maintaining a latent heat of 105.6 J·g^−1^ and providing essential electrical insulation for safe, body-conformable devices. Qu et al. [160] fabricated Janus-type multifunctional PCM membranes through a combination of electrospinning and spraying techniques with a latent heat of 141.4 J·g^−1^, which enabled both active and passive thermal regulation and provided additional EMI shielding functionality (Figure 9c). Gong et al. [161] designed a biomimetic “brick-and-mortar” structured composite film via vacuum filtration. The film offered a latent heat of 136.5 J·g^−1^ and an EMI shielding effectiveness of 43.1 dB, demonstrating preferable capabilities for multi-source driven thermal regulation in advanced wearable applications (Figure 9d).

### 5.2. Energy Storage and Conversion

Thermal energy storage and release are inherent properties of PCMs, while passive thermal storage materials typically rely on external energy sources for operation, limiting their application scope and efficiency [162,163]. Expanding the energy acquisition pathways of PCMs is therefore crucial for advancing their utility in next-generation thermal energy management systems. In recent years, researchers have been dedicated to developing diversified energy conversion technologies [164,165], including the conversion between solar energy, electrical energy, magnetic field energy, and thermal energy, to enrich the multi-source energy capture capabilities of PCMs and achieve multi-level, efficient utilization of energy.

#### 5.2.1. Solar-Thermal/Solar-Thermo-Electric Energy Conversion and Storage

##### Solar-Thermal Energy Conversion and Storage

Solar energy is a clean, renewable energy source, and its efficient utilization has become a research focus. Among the various methods, photo-thermal conversion is considered the most direct and effective approach [41,166]. However, due to the lack of effective photon trapping agents, conventional PCMs typically exhibit poor optical responsiveness, which severely limits their application in solar energy collection, conversion, and storage [117,167]. Introducing highly light-absorbing fillers such as graphene and MXene into the PCM matrix can significantly enhance the light absorption capacity of PCCs, thereby enabling efficient solar energy collection and thermal conversion.

**Figure 10:**
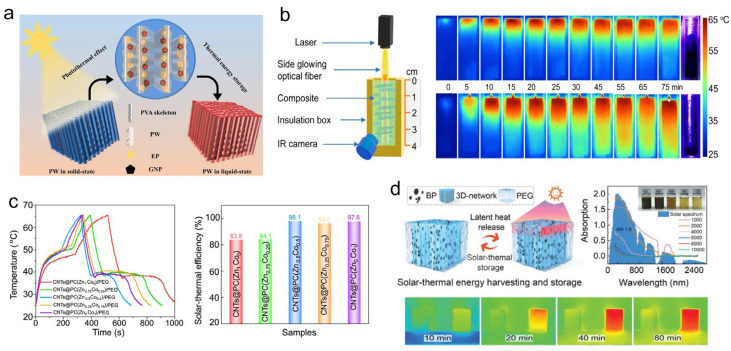
PCCs for efficient solar-thermal conversion and storage. (**a**) Graphene nanoplatelet/PVA/PW composite aerogel demonstrating high light absorption and energy storage density [168]. (**b**) Paraffin–graphene composite with side-glowing optical waveguide fiber for solar-thermal energy storage [169]. (**c**) Cobalt-nanoparticle-loaded carbon heterostructure/PEG composite derived from MOF/CNT precursors, achieving ultrahigh photo-thermal conversion efficiency [172]. (**d**) Black phosphorene/PEG composite aerogel applying the photo-thermal effect of black phosphorene for solar energy utilization [174].

Substantial progress has been made in developing such advanced PCCs. Yang et al. [168] employed graphene nanoparticles (GNPs) as self-assembling units and polyvinyl alcohol as a binder to fabricate graphene aerogel via directed freezing for encapsulating PW. This composite achieved an 85.1% encapsulation efficiency for PW, a heat storage density of 182.9 J·g^−1^, and a remarkable photo-thermal conversion efficiency of 95.2% (Figure 10a). To enhance light utilization, Xue et al. [169] embedded optical fibers into graphene/paraffin composites, creating an internal illumination mode that achieved a conversion efficiency of 94.9% and accelerated the thermal storage rate by 2.2 times compared to traditional surface irradiation (Figure 10b). Li et al. [170] prepared composites of expanded graphite (EG) and pentaerythritol through pressure-induced self-assembly, achieving an ultrahigh thermal conductivity of 33.5 W·m^−1^·K^−1^ alongside a photo-thermal efficiency of 92.7%, facilitating rapid and efficient solar thermal harvesting. Mehrali et al. [171] coated MXene and rGO onto melamine sponge (MS) to obtain a porous aerogel. After impregnation with PW, the composite material exhibited a 66.9% increase in thermal conductivity, a heat storage density of 176.2 J g^−1^, and a light-to-heat conversion efficiency of 93.0%, making it suitable for solar thermal energy storage applications. Tang et al. [172] grew metal-organic frameworks (MOFs) in situ on CNTs, followed by calcination to create cobalt-nanoparticle-loaded carbon heterostructures for PEG encapsulation. The compact heterostructure contributed to a remarkable photo-thermal conversion efficiency of 98.1% (Figure 10c). Liu et al. [173] synthesized an organic/inorganic hybrid polymer for PW encapsulation, resulting in a 600% enhancement in thermal conductivity, an energy density of 180 J·g^−1^, and a conversion efficiency of 93.7%. Wang et al. [174] fabricated black phosphorus aerogel using a cryogenic casting process. After encapsulation with PEG, the composite demonstrated a thermal conductivity of 1.81 W·m^−1^·K^−1^ and an energy density of 103.9 J·g^−1^, effectively leveraging photo-thermal effects for solar energy utilization (Figure 10d).

##### Solar-Thermo-Electric Energy Conversion and Storage

The integration of efficient solar-thermal energy storage with stable photo-thermo-electric conversion presents a significant challenge for contemporary energy systems. Solar thermoelectric generator (STEG) devices, when coupled with PCMs, offer a promising solution to mitigate the intermittency and weather dependence of solar radiation [175,176,177], thereby enabling stable and persistent power output. A primary bottleneck, however, lies in the simultaneous optimization of thermal conductivity, energy storage density, and leakage resistance within the PCM component to ensure the practical application efficacy.

**Figure 11:**
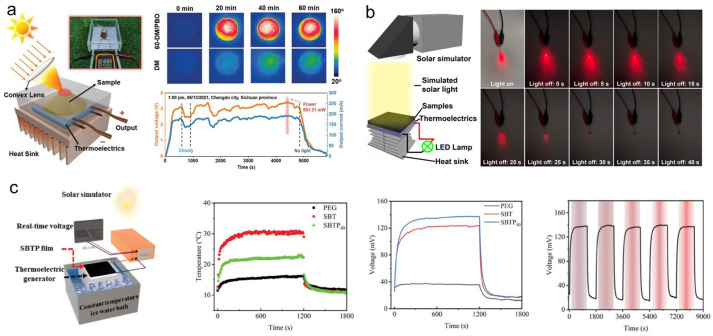
Application of PCCs in STEG systems for solar-thermal-electric conversion. (**a**) Poly(p-phenylene benzobisoxazole) fiber/mannitol (PBO/mannitol) composite demonstrating high power density output [178]. (**b**) Graphene aerogel/PW composite enabling sustained power generation even under dark conditions [180], (**c**) Crosslinked bacterial cellulose/carbon nanotube/PEG composite for solar-thermal energy conversion, storage and utilization [184].

Significant research efforts have been directed toward developing PCCs suitable for STEG systems. Fu et al. [178] engineered a composite using poly(p-phenylene benzobisoxazole) (PBO) fibers and mannitol, which achieved a high thermal conductivity of 22.38 W·m^−1^·K^−1^ and an energy storage density exceeding 90 J·g^−1^. Integration with a STEG device yielded an output voltage of 3.41 V and a power density of 198.7 W·m^−2^ (Figure 11a). Liu et al. [179] implemented an expanded graphite/paraffin wax composite in the STEG, achieving an output voltage of 2.697 V. Shu et al. [180] fabricated graphene aerogels using pre-oxidized polyacrylonitrile (OPAN) and graphene oxide (GO) via unidirectional freeze-casting and graphitization, followed by PW encapsulation. The composite showed a thermal conductivity of 4.36 W·m^−1^·K^−1^ and 99.7% latent heat retention. Under 5 kW·m^−2^ irradiation, the STEG device generated 1181 mV and sustained power delivery to LEDs in the dark (Figure 11b). Yu et al. [181] constructed diamond/cellulose aerogels via ice-templating for eicosane encapsulation, resulting in a composite with 1.53 W·m^−1^·K^−1^ thermal conductivity and 203.5 J·g^−1^ energy density. The STEG coupling delivered an output of 149.5 mV and 59.6 mA. Cai et al. [182] utilized a loofah sponge template to create carbon-based porous materials for PW encapsulation. The composite achieved 84% photo-thermal efficiency and 52 dB EMI shielding, and its integration with a STEG yielded a maximum output of 2.2 V. Shu et al. [183] developed oriented graphene/cellulose aerogels (GCA) for PW encapsulation, achieving a thermal conductivity of 15.9 W·m^−1^·K^−1^ and 98% energy density retention. The STEG device produced an output voltage of 823.2 mV under 5 kW·m^−2^ irradiation. Wang et al. [184] synthesized a crosslinked bacterial cellulose/carbon nanotube/PEG (BC/CNT/PEG) phase change membrane via a one-pot method. The membrane exhibited a thermal conductivity of 0.45 W·m^−1^·K^−1^ and an energy density of 145.1 J·g^−1^. Under 3 kW·m^−2^ irradiation, the coupled STEG produced an output voltage of 423 mV and a power density of 30.26 W·m^−2^ (Figure 11c). Yang et al. [185] fabricated a flexible composite comprising natural rubber, MXene, and PW microcapsules, which exhibited superior thermal conductivity, mechanical strength, and light absorption. When integrated into a STEG, it generated an output voltage of 410 mV.

#### 5.2.2. Electro-Thermal Energy Conversion and Storage

Electro-thermal conversion technology based on PCMs offers a viable strategy for intelligent energy management by storing thermal energy during off-peak electricity periods and releasing it during peak demand [186,187,188]. This “peak load shifting” capability contributes to optimized power grid operation and enhanced overall energy utilization efficiency.

**Figure 12:**
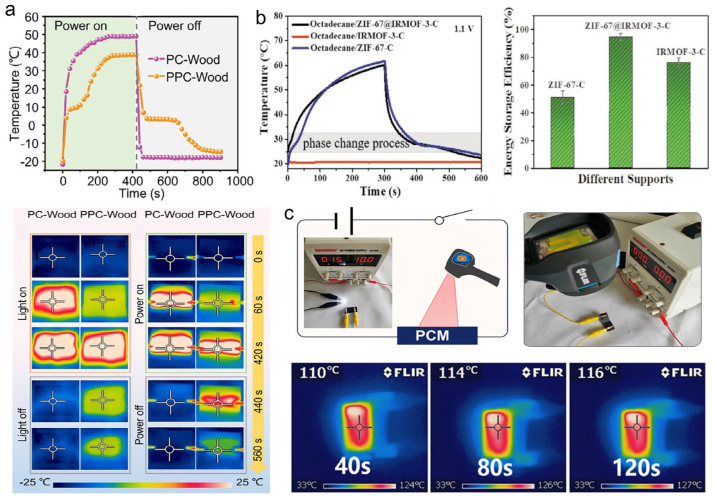
Application of PCCs in electro-thermal conversion and storage systems. (**a**) CNT/balsa wood/PCM composite demonstrating high electro-thermal conversion and storage efficiency [188], (**b**) Carbon nanotube/cobalt porous carbon/octadecane composite derived from MOF carbonization, achieving high efficiency at low operating voltage [193]. (**c**) Aramid nanofibers/MXene hybrid PCM film enabling uniform and rapid electro-thermal conversion [196].

Research in this area has yielded composites with promising performance. Cao et al. [189] encapsulated eicosane within vertically aligned CNT arrays grown via CVD. The composite achieved rapid thermal storage under an ultralow driving voltage of 1.0 V, demonstrating high electro-thermal conversion efficiency. Sheng et al. [188] prepared superhydrophobic CNT/balsa wood with phase change materials, and the composite exhibited a electro-thermal conversion efficiency up to 96.7% under low-temperature conditions (Figure 12a). Yan et al. [190] fabricated expanded graphite (EG)/PEG composites through physical adsorption, attaining a thermal conductivity of 2.48 W·m^−1^·K^−1^ and an energy density of 161.4 J·g^−1^. The electro-thermal performance was found to increase monotonically with higher EG content. Liu et al. [191] developed a composite by modifying nanocellulose with chitosan for PEG encapsulation, which yielded an energy density of 158.3 J·g^−1^ and a high electro-thermal conversion/storage efficiency of 92.3%. Zhang et al. [192] encapsulated PEG within a porous biocarbon matrix, achieving a thermal conductivity of 0.585 W·m^−1^·K^−1^ with 85.7% enthalpy retention and an excellent electro-thermal response. Wang et al. [193] prepared CNT/cobalt porous carbon supports through the carbonization of MOF-5/ZIF-67 hybrids. After impregnation with octadecane, the composite demonstrated a high conversion/storage efficiency of 94.5% at a low operating voltage of 1.1 V (Figure 12b). Wang et al. [194] constructed hybrid carbon aerogels using GO- and MOF-derived carbon, followed by lauric acid encapsulation. The resulting composite exhibited a thermal conductivity of 1.35 W·m^−1^·K^−1^ and an energy density of 140 J·g^−1^, maintaining an electro-thermal efficiency above 90% at a 2.2 V input. Lin et al. [195] utilized carbonized plant straw to fabricate 3D carbon aerogels for PEG encapsulation. The composite achieved an energy density of 185 J·g^−1^. In this system, the grid-structured carbon aerogel acted as an efficient electro-thermal converter, while the lattice-confined PEG functioned as the thermal storage unit, enabling uniform and rapid electro-thermal conversion at low voltages. Tan et al. [196] encapsulated PCMs with aramid nanofibers/MXene hybrid shells, and the obtained HD@ANF/MXene PCM film exhibited a latent heat of 110 J·g^−1^. When applying an external field voltage of 10 V, the electro-thermal storage efficiency of composite reached approximately 96.23% (Figure 12c).

#### 5.2.3. Magneto-Thermal Energy Conversion and Storage

Magneto-thermal energy conversion utilizes magnetic induction materials that generate heat under alternating magnetic fields via mechanisms such as Brownian or Néel relaxation. The incorporation of PCMs with these magnetic matrices enables efficient conversion of magnetic energy into storable thermal energy, creating a unique pathway for controlled heat generation and storage [197,198,199,200,201].

**Figure 13:**
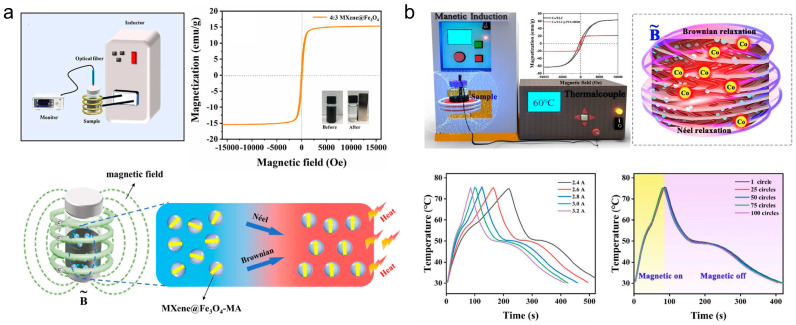
Application of magneto-thermal PCCs. (**a**) Fe_3_O_4_-MXene/myristic acid composite demonstrating rapid heating response [204]. (**b**) ZIF-derived arrayed carbon/PEG composite showing excellent cycling stability [207].

Significant advancements have been made in the design of such magneto-responsive PCCs. Tang et al. [202] modified graphene nanosheets with magnetic Fe_3_O_4_ nanoparticles and compounded them with PEG. The resulting composite achieved a magneto-thermal conversion efficiency of 41.7%, which could be tuned by modulating the magnetic field intensity. Wang et al. [203] combined Fe_3_O_4_@SiO_2_ core–shell particles with PEG, developing composites with exceptional magneto-thermal response suitable for precise temperature regulation in magnetothermotherapy. Gao et al. [204] achieved in-situ anchoring of magnetic Fe_3_O_4_ on MXene surfaces, followed by myristic acid impregnation. The composite delivered an energy density of 144.1 J·g^−1^ and rapidly reached 50 °C under a 1.05 MHz, 10 A magnetic field (Figure 13a). Tao et al. [205] encapsulated PW within polypyrrole/Fe_3_O_4_-functionalized hollow kapok fiber aerogels. The composite exhibited a thermal conductivity of 1.06 W·m^−1^·K^−1^ and an energy density of 161.4 J·g^−1^, achieving rapid heating to 45 °C under an alternating magnetic field (1.3 MHz, 2 A). Chao et al. [206] blended porous carbonized wood powder with Fe_3_O_4_-modified graphene nanosheets and PEG, creating a composite that maintained an energy density beyond 95 J·g^−1^, demonstrating effective magneto-thermal conversion capability. Chen et al. [207] synthesized ZIF-derived 1D-2D bridged-array carbon for PEG encapsulation. The composite demonstrated a saturation magnetization of 21.1 emu·g^−1^ and rapid heating to 75 °C under 3.2 A alternating current fields, along with exceptional magneto-thermal cycling stability (Figure 13b). Xu et al. [208] employed MOF-derived magnetic cobalt-graphitized carbon to encapsulate octadecanol. The composite material achieved a heat storage density of 131 J g^−1^ and could be heated to 75 °C within just 300 s of charging under an alternating magnetic field, showing conversion efficiency proportional to cobalt content.

## 6. Conclusions and Future Research Directions

### 6.1. Conclusions

The development of high-performance PCCs represents a critical pathway toward advanced thermal energy management and efficient energy utilization. The critical challenge in this field lies in achieving an optimal balance between high thermal conductivity and high energy storage density while simultaneously equipping PCCs with multifunctional integration capabilities to meet complex application demands. This review has systematically summarized and discussed recent advancements in the functionalization strategies and multifaceted applications of PCMs, with a particular focus on the construction and role of 3D porous supporting materials. The strategy of encapsulating PCMs within carefully designed 3D frameworks (e.g., metal foams, BN aerogels, carbon-based aerogels, MXene aerogels) has proven highly effective in simultaneously overcoming inherent limitations like low thermal conductivity and leakage while endowing PCCs with high energy storage density and multifunctional properties. These advanced PCCs demonstrate significant potential and have been successfully applied across a broad spectrum of fields, including thermal management applications in electronics, power batteries, buildings, and wearable devices, as well as energy conversion systems utilizing solar-thermal, solar-thermo-electric, electro-thermal, and magneto-thermal conversions. Despite significant progress, future research should prioritize overcoming key challenges in several directions: developing scalable fabrication methods for 3D porous frameworks, advancing from passive storage to active thermal management systems responsive to external stimuli, creating integrated energy conversion devices, and exploring novel applications through innovative techniques like 4D printing. Addressing these challenges will accelerate the development of next-generation PCCs, contributing substantially to global energy conservation, emission reduction, and sustainable energy utilization.

### 6.2. Future Research Directions

The development of high-performance PCCs has demonstrated significant potential for advanced thermal management and energy conversion/storage systems. However, to further advance their practical applications, several key challenges require focused attention in future research:

(1) Scalable fabrication of functional 3D porous frameworks

While 3D porous frameworks effectively address the limitations of pristine PCMs, their synthesis typically involves complex procedures and high energy consumption. Current manufacturing techniques face limitations in achieving large-scale, continuous production due to mold size constraints and process scalability issues. Developing simplified, energy-efficient fabrication strategies that enable precise control over framework orientation through external fields (electric, magnetic, or pressure) represents a crucial research direction to meet diverse application requirements.

(2) Transition from passive to active thermal management

Although PCCs have shown remarkable performance in solar-thermal and solar-thermal-electric conversion systems, their energy storage/release processes remain predominantly passive. Future efforts should focus on developing PCCs with multi-source-driven thermal response characteristics, enabling intelligent thermal management systems that can actively respond to external stimuli. This transition will advance the design of smart composites capable of integrating both active and passive thermal management functionalities.

(3) Integrated solar-thermal-electric conversion systems

The strategic engineering of microstructural and thermoelectric properties within 3D porous frameworks offers promising opportunities for creating self-sufficient PCCs capable of integrated solar-thermal-electric conversion. Such systems could eliminate the need for separate thermoelectric components, thereby simplifying system architecture while enhancing overall energy conversion efficiency. This integrated approach represents an important direction for next-generation energy harvesting technologies.

(4) Innovative manufacturing and functional diversification

Conventional preparation methods need to be revolutionized through advanced manufacturing techniques such as electrospinning and 4D printing. Beyond traditional applications, researchers should explore the unique properties of phase change materials for innovative applications in emerging fields, including infrared stealth technology and information encryption systems. This functional diversification will expand the utility of PCCs beyond conventional thermal and energy applications.

The continued development of PCCs toward scalable fabrication, intelligent thermal management, integrated energy systems, and innovative applications will play a pivotal role in addressing global energy challenges and supporting sustainable development goals.

## Figures and Tables

**Table 1:** Summary of the performances of the PCCs fabricated using different methods.

Fabrication Methods	Supporting Substance	PCM	TC * (W m^−1^ K^−1^)	LH ** (J g^−1^)	Ref.
Melt blend	Copper nanoparticles	Paraffin wax	0.32	157.3	[44]
Solution dispersion	Carbon nanotubes	Paraffin wax	0.419	128.2	[45]
Melt blend	Nano-CuO	Paraffin wax	0.245	152.8	[46]
Melt blend	Graphene nanoplatelets	Paraffin wax	0.7	186.5	[47]
Microencapsulation	TiO_2_/Ti_2_O_3_	Tetracosane	0.509	144.5	[5]
Microencapsulation	Poly(Acrylate)	Bio-based ionic liquids	-	200.93	[56]
Microencapsulation	Polyurethane acrylate	Tetradecane	-	112	[57]
Microencapsulation	Graphene oxide	Paraffin wax	10.4	191.8	[58]
Porous skeleton encapsulation	Nickel foam	Ester-based cetyl palmitate	1.668	180.9	[73]
Porous skeleton encapsulation	Nickel foam	Polyethylene glycol	1.58	126.3	[74]
Porous skeleton encapsulation	CuNW aerogel	Paraffin wax	0.28	173.2	[75]
Porous skeleton encapsulation	Copper foam	Palmitic acid	5.1	174.7	[76]
Porous skeleton encapsulation	Copper foam	Paraffin wax	6.7	116.1	[77]
Porous skeleton encapsulation	BN@Fe_3_O_4_/PVA skeleton	Polyethylene glycol	1.84	121.1	[87]
Porous skeleton encapsulation	BN aerogel	Paraffin wax	-	183	[88]
Porous skeleton encapsulation	BN/GO aerogel	Polyethylene glycol	2.94	147.5	[89]
Porous skeleton encapsulation	BC/BN skeleton	Polyethylene glycol	3.26	137.4	[90]
Porous skeleton encapsulation	rGO aerogel	Lauric acid/myristic acid eutectic	-	124.6	[104]
Porous skeleton encapsulation	Graphite nanosheet/gelatin aerogel	Paraffin wax	3.75	146.4	[100]
Porous skeleton encapsulation	c-GA/MF network	Polyethylene glycol	1.32	167.8	[101]
Porous skeleton encapsulation	Graphene/carbide aerogel	Polyethylene glycol	4.85	149.7	[103]
Porous skeleton encapsulation	Carbon fiber skeleton	Polyethylene glycol	23.1	62.0	[105]
Porous skeleton encapsulation	Graphite nanoplatelet network	Stearic acid	35.0	122.7	[106]
Porous skeleton encapsulation	Graphene foam	Paraffin wax	2.28	160.9	[110]
Porous skeleton encapsulation	Graphene aerogel	Tetradecanol	4.54	206.1	[112]
Porous skeleton encapsulation	MXene/graphene hybrid aerogel	Polyethylene glycol	2.44	129.3	[109]
Porous skeleton encapsulation	Graphene network	Octadecane	1.67	190	[111]
Porous skeleton encapsulation	Graphene aerogel	D-mannitol	8.80	190	[113]
Porous skeleton encapsulation	Graphitized chitosan/graphene aerogel	Polyethylene glycol	2.90	178.8	[114]
Porous skeleton encapsulation	MXene/polyimide aerogel	Polyethylene glycol	-	167.9	[118]
Porous skeleton encapsulation	MXene/biocarbon skeleton	Paraffin wax	-	215.7	[119]
Porous skeleton encapsulation	MXene/K^+^ aerogel	Paraffin wax	-	261.7	[120]
Porous skeleton encapsulation	Wood-derived aerogel	Polyethylene glycol	0.82	135.5	[121]
Porous skeleton encapsulation	MXene/sodium alginate/carbon nanotube aerogel	Tetradecylamine	-	217.8	[122]
Others	Phenolic resin backbone	Polyethylene glycol	0.365	127.6	[127]
Others	Diatomite	Polyethylene glycol	-	105.7	[128]
Others	Polycaprolactamide	Polyethylene glycol	-	96.2	[129]
Others	BN/Polyurethane	Polyethylene glycol	28.3	101	[130]

* TC: thermal conductivity (W m^−1^ K^−1^); ** LH: phase transition enthalpy (J g^−1^).

## Data Availability

No new data were created or analyzed in this study. Data sharing is not applicable to this article.

## Nomenclature

**Abbreviation**	**Expansion**
PCM	Phase change material
PCC	Phase change composite
TES	Thermal energy storage
3D	Three-dimensional
Cu	Copper
CNT	Carbon nanotube
PW	Paraffin wax
CNF	Carbon nanofiber
GNP	Graphene nanosheet
Al_2_O_3_	Alumina
Fe_3_O_4_	Ferroferric oxide
GO	Graphene oxide
SiO_2_	Silicon dioxide
TiO_2_	Titanium dioxide
rGO	Reduced graphene oxide
NF	Nickel foam
CuNW	Copper nanowire
BN	Boron nitride
PVA	Polyvinyl alcohol
PEG	Polyethylene glycol
BC	Bacterial cellulose
MF	Melamine foam
GA	Graphene aeroge
CF	Carbon fiber
CVD	Chemical vapor deposition
PAA	Polyacrylic acid
LM	Liquid metal
EG	Expanded graphite
